# tasiRNA-ARF Pathway Moderates Floral Architecture in *Arabidopsis* Plants Subjected to Drought Stress

**DOI:** 10.1155/2014/303451

**Published:** 2014-08-26

**Authors:** Akihiro Matsui, Kayoko Mizunashi, Maho Tanaka, Eli Kaminuma, Anh Hai Nguyen, Maiko Nakajima, Jong-Myong Kim, Dong Van Nguyen, Tetsuro Toyoda, Motoaki Seki

**Affiliations:** ^1^Plant Genomic Network Research Team, RIKEN Center for Sustainable Resource Science, 1-7-22 Suehiro-cho, Tsurumi-ku, Yokohama, Kanagawa 230-0045, Japan; ^2^National Institute of Genetics, 1111 Yata, Mishima, Shizuoka 411-8540, Japan; ^3^National Key Laboratory of Plant Cell Biotechnology, Agricultural Genetics Institute, Vietnamese Academy of Agricultural Science, Tu Liem, Hanoi, Vietnam; ^4^Integrated Database Unit, Advanced Center for Computing and Communication (ACCC), RIKEN, 2-1 Hirosawa, Wako, Saitama 351-0198, Japan; ^5^Kihara Institute for Biological Research, Yokohama City University, 641-12 Maioka-cho, Totsuka-ku, Yokohama, Kanagawa 244-0813, Japan; ^6^Core Research for Evolutional Science and Technology, Japan Science and Technology, 4-1-8 Honcho, Kawaguchi, Saitama 332-0012, Japan

## Abstract

In plants, miRNAs and siRNAs, such as transacting siRNAs (ta-siRNAs), affect their targets through distinct regulatory mechanisms. In this study, the expression profiles of small RNAs (smRNAs) in *Arabidopsis* plants subjected to drought, cold, and high-salinity stress were analyzed using 454 DNA sequencing technology. Expression of three groups of ta-siRNAs (TAS1, TAS2, and TAS3) and their precursors was downregulated in *Arabidopsis* plants subjected to drought and high-salinity stress. Analysis of ta-siRNA synthesis mutants and mutated *ARF3*-overexpressing plants that escape the tasiRNA-ARF target indicated that self-pollination was hampered by short stamens in plants under drought and high-salinity stress. Microarray analysis of flower buds of *rdr6* and wild-type plants under drought stress and nonstressed conditions revealed that expression of floral development- and auxin response-related genes was affected by drought stress and by the *RDR6* mutation. The overall results of the present study indicated that tasiRNA-ARF is involved in maintaining the normal morphogenesis of flowers in plants under stress conditions through fine-tuning expression changes of floral development-related and auxin response-related genes.

## 1. Introduction

In order to adapt and survive the exposure to biotic and abiotic stress, plants have evolved various molecular responses for fine-tuning the control of adaptive responses that involve posttranscriptional regulatory mechanisms, as well as epigenetic and posttranslational modifications [1, 2]. Recent genome-wide transcriptome analyses using tiling arrays and next generation sequencing (NGS) have revealed a large number of stress-responsive noncoding RNAs (ncRNAs) [3–5].

Several small RNAs (smRNAs), such as miRNAs and siRNAs, were shown to function in development and stress responses in plants [6–9]. In plants, smRNAs exhibit a high level of complexity in their biogenesis and function. At the moment, smRNAs are classified into microRNAs (miRNAs) and three classes of small interfering RNAs (siRNAs) [6–9]. Transacting siRNAs (ta-siRNAs) are derived from* TAS* ncRNAs that are targeted by miR173 or miR390 [10–14]. Double-stranded RNAs (dsRNAs) are generated from cleaved ncRNAs by RNA-dependent RNA polymerase 6 (RDR6) and dsRNAs are processed into 21nt ta-siRNAs.* ARF2*,* ARF3 (ETT)*, and* ARF4* were demonstrated to be targets of* TAS3 *ta-siRNA (tasiRNA-ARF) [10–14].

In the present study,* Arabidopsis* deep smRNA sequencing was used to identify novel roles for smRNAs in abiotic stress response and it was discovered that ta-siRNAs and their precursors (*TAS1, TAS2, *and* TAS3*) were downregulated by drought and high-salinity stress treatments. Analysis of ta-siRNA synthesis mutants subjected to drought and high-salinity stresses revealed a short stamen phenotype and changes in the expression of floral development-related and auxin response-related genes, which was enhanced by the* RDR6 *mutation. These results demonstrate that the tasiRNA-ARF pathway functions in maintaining normal flower morphogenesis under environmental stress.

## 2. Materials and Methods

### 2.1. * *454 Sequencing of Small RNAs

Two-week-old wild-type plants (*Arabidopsis thaliana* ecotype Columbia), grown on MS medium [3], were transferred to drought, cold (4°C), and high-salinity (250 mM NaCl) stressas previously reported [3]. The treated plants were harvested hourly from 1 to 10 hrs after the treatment was initiated. The 1–5 hr stress-treated samples and the 6–10 hr stress-treated samples were pooled into two groups. Total RNAs were prepared using an ISOGEN kit (Nippon Gene) and precipitated with 2 M LiCl and equal volume of ethanol. RNAs were resuspended in RNase-free water at 65°C and extracted twice with an equal volume of phenol for 30 min on ice. The RNAs were then precipitated with 2 M LiCl and equal volume of ethanol. They were resuspended in RNase-free water at 65°C and extracted twice with an equal volume of phenol for 30 min on ice and precipitated again by adding 1/10 volume of 3 M sodium acetate and 3 volume of ethanol. Subsequently, 17–30 nt smRNAs were extracted using flashPAGE (Life Technologies). A cDNA library was then constructed using a small RNA Cloning Kit (Takara). First, smRNAs were ligated with a 5′ adapter F: and a 3′ adapter to generate cDNAs. The cDNAs were electrophoresed in 8 M urea and 7.5% acrylamide gel, and 60–80 nt cDNAs were recovered. The cDNAs were amplified by 12–15 cycles of PCR and subjected to 454 DNA sequencing according to the manufacturer's instructions. To eliminate RNA degradation fragments, the number of RNA sequences was normalized against the total number of miRNA sequences obtained. The normalized number of smRNAs was then subjected to data analysis. Data sets of 454 sequencing are available in DDBJ (http://www.ddbj.nig.ac.jp/index-e.html) under the accession number AB948670-AB967973.

### 2.2. Stress Treatments Applied to tasiRNA-ARF Pathway-Related Mutants

*rdr6-15 *(SAIL_617)*, ago7 *(SALK_095997)*, sgs3 *(SALK_039005)*, dcl4-2 *(GK-160G05)*, ARF3pro;ARF3 *[15]*, ARF3pro:ARF3mut *[15],* arf3* (*ett-15*) [10],* arf4-2* (SALK_070506), and wild-type* Arabidopsis* plants were grown for two weeks in pots containing 30 g of vermiculite soil. The drought stress treatment consisted of subjecting the two-week-old plants to water depletion for one week. After the one-week period, the watering of plants was then reinitiated. The high-salinity stress treatment consisted of watering three-week-old plants that started to bolt, with 100 mM NaCl-containing water for five days.

### 2.3. RNA Extraction

Total RNA was extracted with a Plant RNA Isolation Reagent (Life Technologies) and treated with DNase I (Life Technologies). The RNAs were then subjected to RT-quantitative PCR (RT-qPCR), microarray, and Northern analyses.

### 2.4. RT-qPCR Analysis

cDNAs were prepared from 1 *μ*g of total RNA using Superscript III (Life Technologies). The target RNA concentration was obtained by measuring 1/10 of the cDNA using an ABI Prism 3100 (Life Technologies) and SYBR Premix Ex Taq II kit (Takara). For detecting small RNAs, a SYBR Advantage qPCR Kit (Takara) was used. The relative expression was calculated using the delta-delta CT method. U2 and* ACT2 *for smRNA and mRNA, respectively, were used as a reference gene for normalization. Three independent biological replicates were used in all of the RT-qPCR analyses.

### 2.5. Microarray Analysis

Two-week-old* rdr6 *mutants and wild-type plants were subjected to a drought stress treatment consisting of withholding water for one week. Microarray experiments using flower buds subjected to a drought stress or nonstressed treatment were carried out according to the manufacturer's (Agilent) preferred protocol using three biological replicates [16]. Fluorescent-labeled cRNAs were prepared from each total RNA sample using a Low Input Quick Amp Labeling Kit and were then hybridized to an Agilent* Arabidopsis* V4 microarray. The microarrays were scanned using an Agilent DNA Microarray Scanner G2539A ver. C. 75 percentile normalization was performed for the signals generated by the microarray probes according to the Agilent data analysis protocol. For microarray analysis, R program ver. 2.12.1 was used. Significant differentially expressed genes were identified by 2-way ANOVA analysis (FDR < 0.075) [17, 18]. The data set derived from the microarray analysis is available in GEO (http://www.ncbi.nlm.nih.gov/geo/info/linking.html) under the accession number GSE57174.

## 3. Results

### 3.1. RNA Sequencing of* Arabidopsis* Small RNAs in Plants under Abiotic Stress

Two-week-old wild type* Arabidopsis* plants were subjected to drought, cold, and high-salinity stress as described in Section 2. Six smRNA libraries were prepared from pooled samples of stress-treated plants and one pooled sample from nonstressed control plants using 454 DNA sequencing technology as described in Section 2. A total of 480,343 reads were obtained from these seven libraries. The smRNA sequences (17–30 nt) were used for further analysis. After the smRNA sequence data were assembled to unique sequences in each library and they were mapped to the* Arabidopsis *genome, resulting in 59,284 sequences that were perfectly matched to at least one locus (Table 1). The sequences represented 12,028 unique signatures in whole 7 libraries. Approximately 39% (4,681) of the unique signatures were represented by a single sequence. The smRNAs were classified based on their mapped genomic position. The composite profiles of the types of identified smRNAs were different in each stress treatment (Supplemental Figure 1; Supplementary Material available online at  http://dx.doi.org/10.1155/2014/303451). The percentile of smRNAs mapped to the sense strand of AGI code genes was higher in drought-stress and high-salinity-stress treated samples than in the nonstressed control sample, suggesting that mRNA degradation occurs preferentially under these stress conditions.

### 3.2. Identification of Stress-Responsive miRNAs

Deep RNA sequencing analysis of smRNAs identified signatures of various miRNAs, including those previously reported to be stressresponsive miRNAs in* Arabidopsis* (Supplemental Figure 2). Expression of miR169 was downregulated in response to drought stress (Supplemental Figure 2). Downregulation of miR169 by drought has also been previously reported and demonstrated to be required for the acquisition of drought stress tolerance [19]. Expression of miR156 and miR319 was upregulated by salinity stress and miR408 expression was upregulated by cold stress (Supplemental Figure 2). These results are also consistent with a previous report [20].

### 3.3. Accumulation of ta-siRNA Expression

**Figure 1:**
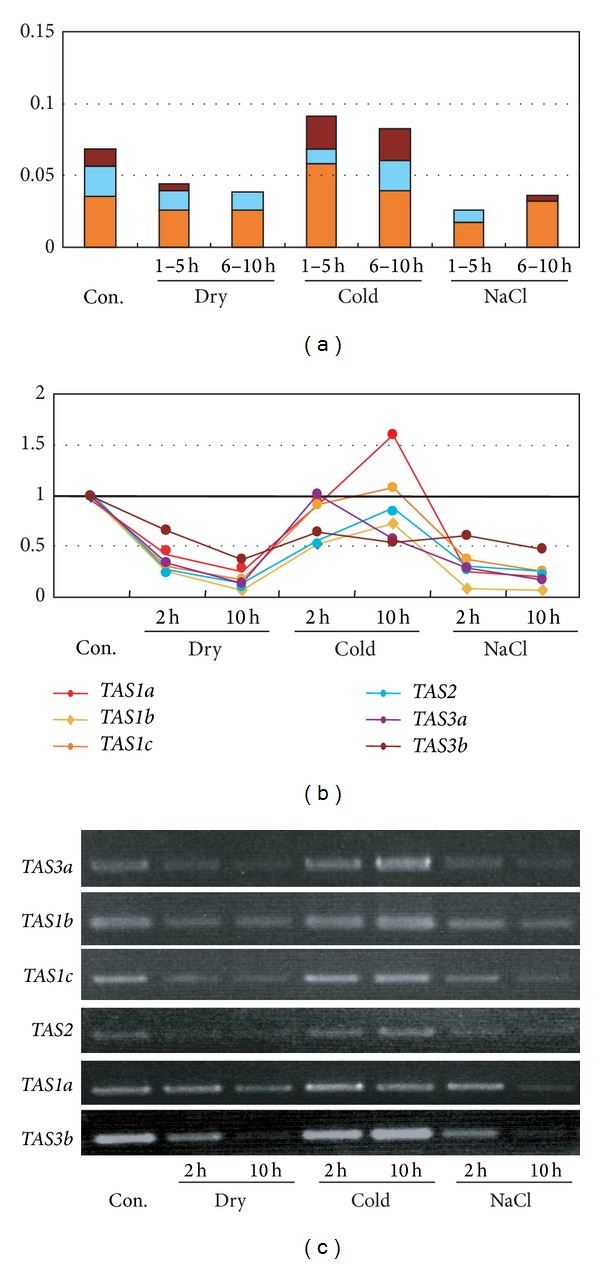
Downregulation of ta-siRNAs and TAS precursors in plants under drought and high-salinity stress. (a) Relative number of ta-siRNA sequences obtained by 454 DNA sequencing of cDNA libraries prepared from small RNAs. Orange, blue, and red boxes represent the relative number of* TAS1 *ta-siRNA,* TAS2 *ta-siRNA,and* TAS3 *ta-siRNA, respectively. (b) Relative expression of* TAS* precursors was analyzed using previous data from a tiling array [3]. Two-week-old* Arabidopsis* plants were subjected to drought (2 hr, 10 hr), cold (2 hr, 10 hr), and high-salinity (2 hr, 10 hr) (see Section 2). (c)PCR primer sets (Supplemental Table 1) spanning miRNA cleavage site were designed to determine the expression profiles of* TAS* precursors. RNA samples were isolated from drought (2 hr, 10 hr), cold (2 hr, 10 hr), and high-salinity (2 hr, 10 hr) treated wild-type plants and nonstress controls. Expression of* TAS* precursors was analyzed by semiquantitative RT-PCR.

A further analysis of smRNA sequences identified in the present study revealed that the number of ta-siRNAs was reduced in response to the stress treatments when compared to the number of ta-siRNAs identified in the nonstressed control (Figure 1(a) and Supplemental Figure 2). The precursors of tasiRNA are transcribed as ncRNAs and processed into siRNAs after a miRNA cleavage event [10–14]. An analysis of* Arabidopsis* expression profiling data obtained from a previous study utilizing a tiling array [3] indicated that the expression of the* TAS1/2/3* family was downregulated under drought and high-salinity stress (Figure 1(b)). Semiquantitative RT-PCR analysis, using primer sets that span the miRNA cleavage sites, showed that the expression of* TAS1/2/3 *precursors decreased under drought and high-salinity stress (Figure 1(c)). These results are consistent with the ta-siRNA expression profiling data obtained from smRNA sequencing. Collectively, the data indicate that abiotic stress signaling regulates ta-siRNA production through transcriptional regulation of their precursors.

**Figure 2:**
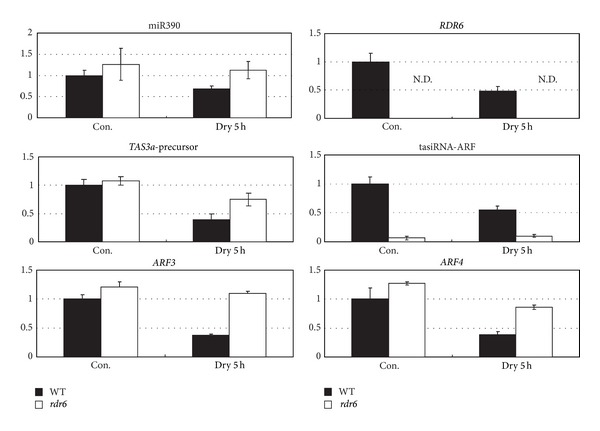
Expression profiles of miR390,* TAS3* precursor,* ARF3,* and* ARF4* in plants under drought stress. Expression profiles of tasiRNA-ARF pathway-related genes were analyzed in* rdr6* mutants and wild-type plants subjected to a 5 hr drought stress and nonstressed conditions by RT-qPCR. The bar graphs indicate relative expression compared to* ACT2*. Values represent the mean and standard deviation of three experiments.

It is known that ta-siRNA-ARF guides the cleavage of* ARF3* and* ARF4 *mRNAs [10, 11, 14, 15]. Therefore, the accumulation of* TAS3 precursor*, ta-siRNA-ARF,* MIR390, ARF3,* and* ARF4 *in wild-type plants and a ta-siRNA synthesis mutant,* rdr6,* in response to a five-hour-drought stress treatment was measured using RT-qPCR in order to better understand the function of ta-siRNA in abiotic stress response (Figure 2). Expression of the* TAS3a* precursor and* RDR6* was downregulated under drought stress (Figure 2). The TAS3a expression data is consistent with the tiling array data (Figure 1(b)) [3] and results obtained by semiquantitative RT-PCR (Figure 1(c)). Expression of* ARF3* and* ARF4* was downregulated under drought stress in wild-type plants but the level of downregulation in* rdr6* mutants was much less (Figure 2). These results suggest that effective downregulation of ARF3/ARF4 mRNAs under drought stress occurs by degradation activity of tasiRNA-ARF (Figure 2) and transcriptional repression of ARF3/ARF4 mRNAs (Figure 2) under drought stress. Downregulation of tasiRNA-ARF might function in fine-tuning quantitative expression of ARF3/ARF4 under drought stress.

### 3.4. ta-siRNA Generation-Related Mutants Fail to Self-Pollinate due to Modifications in Flower Architecture

**Figure 3:**
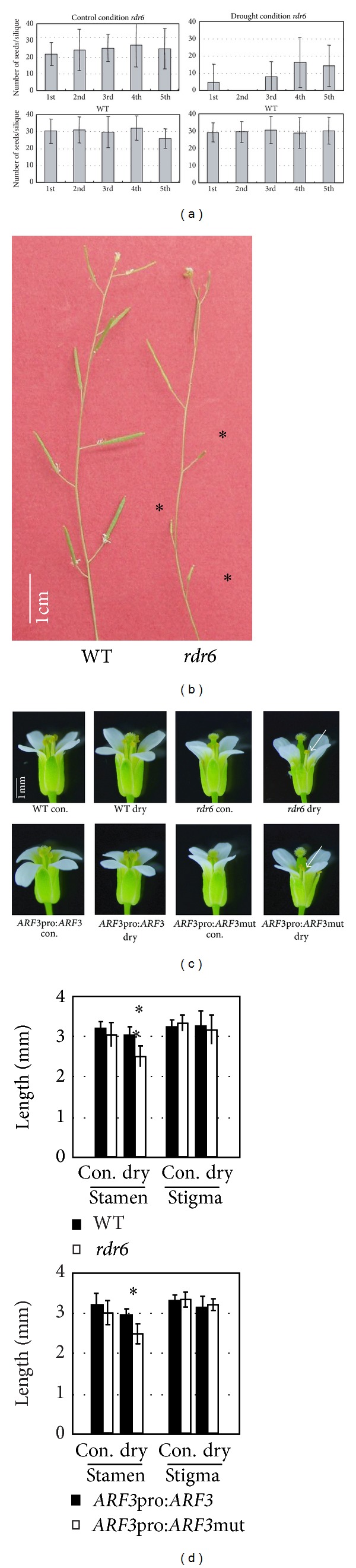
Modification of flower architecture and reduction in seed number in the ta-siRNA biosynthesis mutant,* rdr6, *under drought stress. (a) Two-week-old* rdr6* and wild-type plants were subjected to drought stress treatment for one week and then rewatered. Seed number in the 1st through the 5th siliques was counted (*n* = 12). Siliques were numbered starting at the basal end of the main shoot. (b) Siliques in plants subjected to drought stress followed by rewatering. (c) Floral architecture in drought-stress and nonstressed wild-type,* rdr6*,* ARF3pro:ARF3, *and* ARF3pro:ARF3mut *plants. Flowers in* rdr6* and* ARF3pro:ARF3 *plants have a slightly exerting stigma phenotype under nonstressed conditions. In contrast, flowers in* rdr6* and* ARF3pro:ARF3mut* plants under drought stress exhibit short stamens, as shown as white arrows. (d) Average length of the stigmas and stamens in drought-stressed and nonstressed WT,* rdr6*,* ARF3pro:ARF3, *and* ARF3pro:ARF3mut *plants.

In order to identify the biological function of ta-siRNA under environmental stress, a moderate drought stress was applied to a variety of* Arabidopsis* mutants that are deficient in ta-siRNA biosynthesis (*rdr6, sgs3*,* dcl4,* and* ago7*). The moderate drought stress was applied to two-week-old plants grown in soil by withholding water for one week, resulting in an approximate 20% decrease in soil water content (Supplemental Figure 3(a)). Following the drought treatment, the plants were rewatered. After recovery, wild-type plants produced a number of seeds that was similar to the numbers produced in nonstressed control plants (Figures 3(a) and 3(b)). On the other hand, ta-siRNA mutants, such as* rdr6, sgs3, dcl4,* and* ago7, *produced a lower number of seeds after recovery from the drought stress compared to the numbers produced in nonstressed ta-siRNA mutants (Figures 3(a) and 3(b) and Supplemental Figure 3(b)).

The reproduction failure phenotype was further investigated and it was found that the reproduction failure of* rdr6* plants under drought stress could be rescued by artificial pollination (Supplemental Figure 3(c)). These data suggested that the reproduction failure under drought stress was due to insufficient contact between the anther and the stigma. Abnormal floral architecture was observed in stage 13 flowers of* rdr6* plants, which had shorter stamens relative to the length of the stigma (Figure 3(c)). The length of stigmas and stamens in stage 13 flowers was examined in* rdr6* and wild-type plants under drought stress and nonstressed conditions. Stigma lengthin* rdr6* and wild-type plants was similar in plants under drought stress and nonstressed conditions. In contrast, the length of* rdr6* stamens was shorter in plants under drought stress compared to the length of stamens in wild-type plants under drought stress (Figures 3(c) and 3(d)). Although* rdr6* plants exhibited a tendency to produce slightly shorter stamens, relative to wild-type plants, even in nonstressed conditions, the difference was not statistically significant (Figures 3(c) and 3(d)). These data were consistent with a previous report [21].

It has been demonstrated that tasiRNA-ARF negatively regulates* ARF3* and* ARF4, *both of which are genes that control flower organ identity [22–24]. The ability of tasiRNA-ARF to regulate correct stamen development under drought stress was examined using* ARF3pro:ARF3mut*. This transgenic genotype has a mutated* ARF3* sequence that confers the ability of* ARF3* to avoid tasiRNA-ARF targeted degradation and still translate ARF3 protein [15]. Similar to* rdr6 *plants, the* ARF3pro:ARF3mut* exhibited a short stamen phenotype in plants subjected to drought stress (Figures 3(c) and 3(d)), suggesting that the tasiRNA-ARF pathway has an important role in regulating stamen length under drought stress. Additionally, reproductive failure and the short stamen phenotype were also observed in* rdr6* mutants and* ARF3pro:ARF3mut* plants that were subjected to high-salinity stress (Supplemental Figures 3(d), 3(e), and 3(f)). These results suggest that the tasiRNA-ARF pathway plays a critical role in the regulation of floral organ development under both drought and high-salinity stress.

### 3.5. *RDR6* Functions in the Stabilization of Stress-Dependent Changes in the Expression of Floral Development-Related and Auxin-Related Genes in Plants under Drought Stress

**Figure 4:**
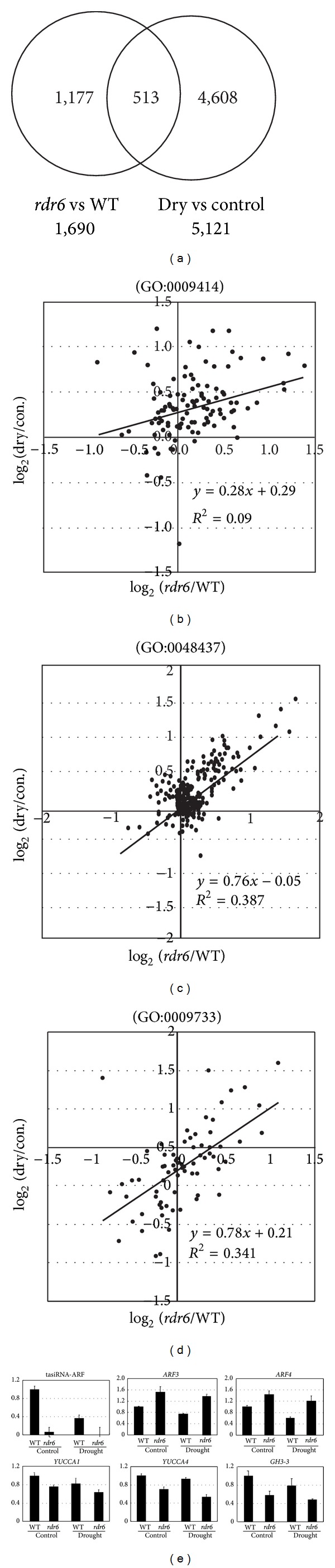
Microarray analysis of flower buds in plants under drought stress. (a) Venn diagram of differentially expressed genes in* RDR6* plants compared to wild-type plants and drought stress-responsive genes. Statistically significant differentially expressed genes were identified based on the following criteria: FDR of 2-way ANOVA (*rdr6* versus WT or drought-stressed versus nonstressed) < 0.075. ((b)–(d)) Scatter plot analysis of genes from different categories of GO terms. Horizontal axis represents log_2_ ratio of (*rdr6* non-stressed +* rdr6* drought)/(WT nonstressed + WT drought). Vertical axis represents log_2_ ratio of (WT drought+* rdr6* drought)/(WT nonstressed +* rdr6* non-stressed). (b) Scatter plot analysis of water deprivation response-related genes. (c) Scatter plot analysis of floral organ development-related genes. (d) Scatter plot analysis of auxin response-related genes. (e) RT-qPCR expression profiles of tasiRNA-ARF,* ARF3*,* ARF4*,* GH3-3*,* YUCCA1, *and* YUCCA4* in flower buds of* rdr6 *mutant and wild-type plants subjected to drought stress or nonstressed control.

Gene expression profiles in flower buds of* Arabidopsis* plants under moderate drought stress and nonstressed conditions were analyzed using a microarray in order to better understand the regulation of the ta-siRNA-mediated network in response to drought stress. Results identified 513 (30.3%) genes whose level of expression was significantly different in* rdr6* plants compared to wild-type plants. The list of genes overlapped with drought stress-responsive genes (Figure 4(a), Supplemental Table 2). No correlation (*R* ^2^ = 0.09) was observed between the expression ratios of drought/control and those of* rdr6*/wild-type for the GO category of water deprivation response-related genes (GO:0009414) (Figure 4(b)).* rdr6 *and wild-type plants showed similar drought stress-responsive expression in the water deprivation response-related genes (Supplemental Figure 4(a)). These results suggest that a similar level of drought stress was applied to* rdr6* and wild-type plants.

Among the differentially expressed genes in* rdr6* plants were a number of floral development-related genes (Supplemental Table 3). Expression levels of the C-class homeotic gene* AGAMOUS*  [25] and* AGAMOUS *downstream genes that promote the development of stigma, style, and medial tissue of ovules, such as* SHATTERPROOF 1* and* SHATTERPROOF 2* [26], and the stigma and stamen identity gene,* SUPERMAN* [27], were upregulated under drought stress and their upregulation was affected in* rdr6 *mutants (Supplemental Table 3). In contrast, expression of the E-class organ identity gene,* SEPALLATA 4 *[28], and the petal identity gene* PETALLOSS *[29] was downregulated in response to drought stress in both wild-type and* rdr6* mutants; however, their expression was significantly lower in* rdr6 *mutants relative to wild-type plants (Supplemental Table 3). These results indicate that the* RDR6* mutation affects organ whorl identity genes and their downstream genes. It seems that tasiRNA-ARF regulation represses central floral organ development and enhances peripheral floral development under drought stress.

To better understand the relationship between drought stress response and the* RDR6* mutation on flower development-related genes (GO:0048437), the expression ratios of drought/control and those of* rdr6*/wild-type were compared. A moderate positive correlation (*R* ^2^ = 0.387) was observed between the drought response and* RDR6* mutation (Figure 4(c)). These data suggest that tasiRNA-ARF is involved in fine-tuning the expression of floral development-related genes in plants subjected to drought stress.

## 4. Discussion

The results of the present study demonstrate that tasiRNA-ARF functions in keeping correct flower architecture which is critical to self-pollination under drought and high-salinity stress. Various abiotic stresses, such as heat, high-salinity, drought, and cold, induce reproductive failure in plants [30–35]. This failure is the result of morphological abnormalities that arise during various stages of floral development. Molecular mechanisms responsible for the abortion of pollen development have been well-characterized [30–35]. Although defects in stamen development in plants under drought, high-salinity, and heat stress have been reported [32, 34, 35], the molecular mechanisms that protect floral development from the adverse effects of abiotic stress, however, remain unclear.

tasiRNA-ARF is required for the normal development of lateral organs, such as leaves, lateral roots, and flowers [14, 21, 36, 37]. For example,* rdr6* mutant exhibits altered stamen and pistil elongation that results in variable seed production and supports the premise that reproduction in* rdr6 *plants is sensitive to growth conditions [22]. Interestingly, a mutation of* RDR6* enhanced self-incompatibility in a transgenic, self-incompatible,* Arabidopsis thaliana *system [38]. These results suggest that tasiRNA-ARF functions as a key mediator for maintaining the correct pattern of flower architecture, as well as their development.* ARF3* and* ARF4* function in central organ identity in flowers and apical-basal patterning defects in the gynoecium [22–24].

**Figure 5:**
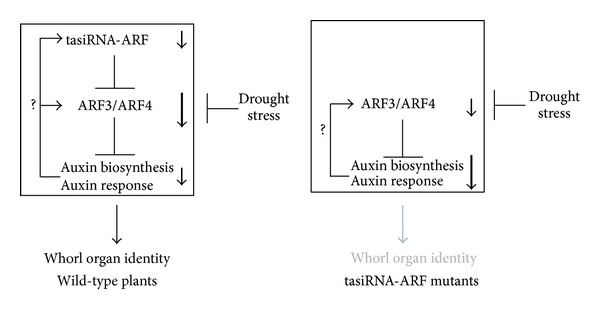
Proposed model for the fine-tuning of whorl architecture in flowers subjected to environmental stress via tasiRNA-ARF. tasiRNA-ARF is required for normal morphogenesis of floral whorl organs in plants subjected to drought stress. tasiRNA-ARF acts by modulating the expression of floral development-related genes in plants subjected to drought stress. Expression of* YUCCA4* is downregulated by drought stress and in the* RDR6* mutation, suggesting that auxin biosynthesis is modulated by tasiRNA-ARF.

*ARF*s regulate the expression of auxin-responsive genes by binding specifically to auxin response elements (AuxRE) [39]. Regarding auxin response-related genes (GO:0009733), a moderate positive correlation was observed between drought stress response and the effect of the* RDR6* mutation (Figure 4(d)). The genes downregulated by both the drought stress treatment and the* RDR6* mutation included the auxin-induced conjugating enzymes,* GH3-2*,* GH3-3*,* GH3-6*,* GH3-10*, and an* auxin-responsive GH3-like protein* (*AT1G48660*) (Supplemental Table 2). In addition, microarray analysis also identified that an expression of auxin biosynthesis-related gene,* YUCCA 4,* was significantly downregulated by drought stress and the* RDR6* mutation (Supplemental Table 2). After conducting a closer study of 4* YUCCA* genes that are mainly expressed in flowers [40],* YUCCA 1* and* YUCCA 4* were downregulated by drought stress and* RDR6* mutation and* YUCCA 2* and* YUCCA 6* were downregulated by* RDR6* mutation (Supplemental Figure 4(b)). The regulation of tasiRNA-ARF and mRNAs of* ARF3, GH3-3*,* YUCCA 1,* and* YUCCA 4* in flower buds was confirmed by RT-qPCR (Figure 4(e)). The previous report showed that expression of an auxin reporter, DR5:GUS, was decreased in* ARF3pro:ARF3mut* plants [41]. These results suggest that the auxin biosynthesis and auxin response were attenuated in floral development by drought stress and loss of tasiRNA-ARF regulation. It was known that auxin signaling was important for floral development. The pin-shaped flower of* yucca1/4* was similar to the flowers produced in* ARF3-* or* ARF4-*overexpressing plants [40]. Experiments utilizing an auxin transport inhibitor indicate that* ARF3* functions as a modulator of auxin response during floral development [42]. There is a possible hypothesis that tasiRNA-ARF controls floral development by maintaining the proper level of auxin signaling under drought stress (Figure 5).

Both tasiRNA-ARF and* ARF3/4* were downregulated under drought stress, suggesting that tasiRNA-ARF are required for quantitative adjustment of* ARF3/4* expression. Positive feedback regulation of auxin signaling might also function in the regulation of these genes (Figure 5). It is known that initiation of lateral roots modulated by positive and negative feedback regulation between tasiRNA-ARF and* ARF2/3/4* through auxin signaling [38, 39].* ARF3* expression was also induced by increased auxin biogenesis through upregulation of* YUCCA 4* in shoot initiation [43]. These previous reports invoke that drought stress affects tasiRNA-ARF regulatory network, but it remained unclear.

To search candidate genes connecting abiotic stress and tasiRNA-ARF regulatory network, microarray coexpression analysis of ARF3 (*P* < 0.01) and ARF4 (*P* < 0.01) was performed. 155 genes coexpressed with* ARF3 *and* ARF4 *involved twenty-five abiotic stress response-related genes, such as* DREB2C *[44],* DWD* (*DDB1-binding WD40 protein*) [45],* ascorbate peroxidase 1* [46],* glutathione S-transferase *[47], and* RD21B* [48] (Supplemental Table 4). Thegenes also included ta-siRNA pathway-related genes, such as* RDR6*,* TAS3*, other* TAS* genes, and ta-siRNA target genes [49]. Sixty-four of the 155 genes possessed an ARF binding motif (tgtctc) [50] in the promoter region within 1kb upstream of the start codon (Supplemental Table 4). These genes may represent candidates that connect tasiRNA-ARF regulatory network and drought stress signaling pathway.

In conclusion, this study demonstrates that tasiRNA-ARF acts as a central modifier, negatively regulating changes in the expression of floral development-related genes in plants under drought and assists in maintaining normal floral morphogenesis.

## Supplementary Material

Supplemental Figure 1. Distribution of smRNAs in abiotic stress-treated plants. smRNA sequences were classified based on where they mapped in the Arabidopsis genome (TAIR8). The circular graphs illustrate the distribution of mapped smRNA sequences obtained from plants subjected to each different treatment. The miRNAs and ta-siRNAs indicate that smRNA sequences are derived from MIRNA loci and TAS loci. Small RNAs mapped to regions coding for proteins were classified into two groups, smRNAs mapped to the sense strand and smRNAs mapped to the antisense strand of protein-coding genes. Transposable element (TE) genes and pseudogenes are AGI coded genes annotated as TEs and pseudo genes, respectively. Intergenic TEs represent smRNAs mapped to the TEs in intergenic regions.
Supplemental Figure 2. Deep sequencing of miRNAs and ta-siRNAs in non-stressed and stress-treated plants. The number of RNA sequences was normalized by dividing by the total number of obtained miRNA sequences. P-values were calculated using an enrichment test on each miRNA fraction, based on their hypergeometric distribution. Asterisk in the table indicates the p-value was lower than 10-3. Previously reported stress- upregulated or downregulated smRNAs are shown in red or blue, respectively.
Supplemental Figure 3. Seed production in ta-siRNA biosynthesis mutants, rdr6, sgs3, dcl4 and ago7, subjected to drought stress, and in rdr6 and ARF3pro:ARFmut mutants subjected to high-salinity stress. (a) Soil water content during the course of the imposed drought treatment. (b) Seed numbers in the 1st silique (n =12). (c) Seed number in the 1st silique of plants subjected to a drought stress after artificial pollination and self-pollination (n =12). (d) Seed numbers in the 1st silique of three-week-old plants grown in soil and treated with 100 mM NaCl for one week (n =12). (e) Short siliques were observed in rdr6 and ARF3pro:ARF3mut plants subjected to high-salinity stress. (f) Short stamens were observed in rdr6 and ARF3pro:ARF3mut plants subjected to high-salinity stress.
Supplemental Figure 4. Scatter plot analysis for drought stress-responsive expression in rdr6 and wild-type plants. (a) Scatter plot analysis for drought stress-responsive expression of water deprivation response-related genes (GO:0009414) in rdr6 and wild-type plants. Horizontal axis represents log2 ratio of (drought / non-stressed) in wild-type plants. Vertical axis represents log2ratio of (drought / non-stressed) in rdr6 mutants. (b) Line graph for expressions of YUCCA genes. Horizontal axis represents normalized intensities of microarray probes targeting YUCCA genes.
Supplemental Table 1. List of primer sequences.
Supplemental Table 2. Differentially expressed genes in flower buds of rdr6 and wild-type under drought stress treatment and untreatment.
Supplemental Table 3. Expression profiles of floral organ development-related genes (GO:0048437)
Supplebmental Table 4. Expression profiles of 155 genes co-expressed with ARF3 and ARF4.

## Figures and Tables

**Table 1:** Number of smRNA-seqs and smRNA loci in each treatment.

Treatment	Number of sequences^a^	Number of loci^b^
Drought		
1–5 h	17,758	4,574
6–10 h	10,367	3,138
Cold		
1–5 h	2,104	1,595
6–10 h	2,869	2,018
High salinity		
1–5 h	10,247	3,645
6–10 h	6,148	2,417
No treatment	9,791	4,162

^a^smRNA sequences were mapped on *Arabidopsis* genome. Sequences of tRNAs, rRNAs snoRNAs, and snRNAs were eliminated. ^b^smRNAs mapped within less than 150 nt distance were grouped as the same loci.
